# Bioburden of postmortem bone tissues with a procurement time exceeding 36 h

**DOI:** 10.1007/s10561-025-10174-3

**Published:** 2025-05-26

**Authors:** Christian Braun, Matthias Löwel, Miriam Heuer, Axel Pruß, Tino Schulz

**Affiliations:** 1 Institute of Legal Medicine Munich (LMU), Nussbaumstrasse 26, 80336 Munich, Germany; 2 DIZG Deutsches Institut für Zell- Und Gewebeersatz gGmbH, Köpernicker Str. 325, 12555 Berlin, Germany; 3 Institute of Transfusion Medicine (Charité Berlin), Luisenstraße 7, 10117 Berlin, Germany

**Keywords:** Tissue donation, Bioburden, Postmortem interval, Sterilization

## Abstract

To qualify for bone donation many criteria must be met. For procurement, two time intervals—a postmortem interval (PMI) of 6 h (hours) until cooling of the deceased and a maximum procurement time of tissues within 24/36 h postmortem are mandatory. Expanding the procurement to donors with a longer PMI would be helpful e.g. to have more time to contact relatives for consent for donation. A loss of biomechanical quality of bone tissues should not be expected in the PMI until procurement, but the question of microbiological contamination remains. Therefore, samples from the iliac crest were procured under standard procurement conditions between 48 and 54 h (n = 14, group 1) and 72–78 h (n = 7, group 2) postmortem (pm). Samples were immediately frozen after procurement at < − 18 °C and sent to the German Institute for Tissue and Cell Replacement (DIZG) for mechanical preparation. Additionally, chloroform treatment was performed at the tissue bank of the Charité Berlin. After each step the samples were refrozen and sent to a contracted microbiological lab for bioburden testing according to the European Pharmacopoeia. Samples were tested after procurement, preparation, and chloroform treatment respectively. As acceptance criterion a maximum amount of 9 × 10^4^ colony forming units per gram (CFU/g) tissue was defined. All samples were within the defined limit with a maximum value of 2.5 × 10^2^ CFU/g. These values are in the range of bone tissues procured under non-strict-aseptic procurement conditions within 24 or 36 h and are within the capacity of the peracetic acid sterilization process used by several tissue banks.

## Introduction

Musculoskeletal tissues (MST) from postmortem donors are in high demand for transplantation purposes. Before the corona pandemic, there was an increase in bone tissue donations, at least in some European regions, but the demand for corresponding transplants could not be met (El Hage et al. 2018).

The corona pandemic resulted in decreasing donation numbers (Börgel et al. 2022). Other factors with the possibility of negatively affecting tissue donation in the future are the demographic change with higher donor age (Hofmann et al. 2022) and the spread of infectious diseases (e.g. West Nile Virus) influenced by climate change (Rios 2009).

But even without these observed changes, many criteria apply to qualify a donor for bone procurement. Beside exclusion due to donor medical history, serological and PCR testing, the EDQM-Guide (Chapter 7 Procurement: 7.4 Procedures) recommends a postmortem interval (PMI) of 6 h until refrigeration (2–8 °C) or cooling (8–15 °C) of the donor body and a maximum procurement time of the tissues within 24 to 36 h postmortem depending on sterilization method (Doerr 2022). The EDQM-Guide also states that it may be possible to extend the PMI up to 48 h, if processing has been validated to guarantee quality and microbiological safety (Doerr 2022).

These narrow time intervals are significant challenges for MST donation, as the next of kin must be successfully contacted and given the time for a decision, with the potential of donor loss. To give an example in our forensic setting (Wulff et al. 2014), in 2022 we were unable to identify or contact the next of kin in time in 50 cases, accounting for 40% of potential MST donors.

Because of the criticality of time restraints, a longer postmortem procurement period would be helpful to procure tissues from more feasible donors if a validated sterilization process is used.

To extend the postmortem interval until tissue procurement, two aspects must be kept in mind: Potential changes in the biomechanical properties (i.e. quality) of tissues and the possibility of bacterial or fungal contamination.

There are several studies concerning the changes in tissues like pericardium (Bernd 2008), fascia lata (Storch 2002) and dura mater (Engelhardt 2000) that show no loss of biomechanical quality (e.g. elasticity, tear resistance) up to 14 days (Bernd 2008) postmortem and longer (Storch 2002; Engelhardt 2000). For bone tissue, loss of water and degradation of collagen may change the biomechanical properties postmortem (Wescott 2019), but forensic studies have shown that these changes do not affect the properties of bone fractures for at least 28 days in a porcine model (Wieberg and Wescott 2008) and up to 60 days in a human model (Skinner et al. 2023). Therefore, biomechanical changes of these slowly metabolizing tissues in a cooled body affecting transplant quality in a PMI of up to 72 h before processing seem unlikely.

However, the risk of bacterial or fungal contamination of tissues is well known (Eastlund 2006). Several studies demonstrated a higher contamination rate with longer postmortem intervals until procurement (Brubaker et al. 2016; Paolin et al. 2017a; Bohatyrewicz et al. 2006; Vehmeyer et al. 2002). Therefore, the critical parameter is the microbiological load of the tissue which must be eliminated by a sterilization process. The validation of peracetic acid/ethanol sterilization (v. Versen et al. 1993) has shown that an efficient reduction of 6 log^10^ CFU/g for the microorganisms *Staphylococcus aureus*, *Enterococcus faecium*, *Pseudomonas aeruginosa*, *Bacillus subtilis*, *Clostridium sporogenes*, *Mycobacterium terrae* and *Candida albicans* was achieved after just 2 h of exposure (Pruss et al. 2001). Transplants sterilized with this method were successfully applied without any incidences of disease transmission (Pruss et al. 2002).

To cope with this problem, it is advisable to perform a bioburden determination of the tissues and use a validated sterilization process with further sterility testing after sterilization in accordance with the European Pharmacopoeia, Chapter 2.6.1. (European Pharmacopoeia 2022).

Under the conditions described above, this study is intended a) to quantify the contaminating bioburden of bone tissues procured at 48- and 72-h postmortem, and b) to assess whether this microbial load is within the capacity of the peracetic sterilization process used. If this is the case, an extension of the PMI until procurement could be feasible.

This study was carried out considering German laws and by-laws as well as guidance of the competent authority (Paul-Ehrlich-Institute) concerning tissue donation (e.g. postmortem intervals, contact to the next of kin, procurement of tissues).

## Material and methods

### Bone donors and sample procurement

For this study, we procured the right iliac crest from 14 non-heart beating donors (NHBD) within 48–54 h and from 7 NHBD donors within 72–78 h postmortem according to standard operating procedures for bone procurement. A control group with 4 NHBD donors and procurement within 36 h was also included.

All procedures were performed in a forensic setting by the same trained and experienced staff. After procurement, reconstructive measures were taken, with the only visible change in form of a short autopsy suture.

All donors were cooled within 6 h after death at 5–7 °C and were of adult age. Contraindications were known drug abuse, sexually transmitted and tropical diseases, suspicion of Creutzfeldt-Jacob disease, heavy trauma to the pelvis and obvious putrefaction. Blood samples from all donors were taken within 24 h after death and were tested for Hepatitis B, C and HIV (serology and nucleic acid amplification).

Before procurement, the skin was disinfected, and sterile drapes were used to protect the sampling area. The procedure was then carried out using sterile instruments. The ilium was exposed as per usual for hemipelvis procurement. Then, utilizing a surgical mallet and osteotome, the iliac crest was removed so that a piece of tricortical ilium measuring approximately 12 to 20 cm^3^ was obtained. The bone sample was then transferred to a sterile disposable container and frozen at < − 18 °C.

The iliac crest samples were transported to the tissue bank of the DIZG. After thawing 3 Os ilium cubes (approx. 2 × 1.5 × 1 cm) were cut from each sample. 

One sample cube was then transferred immediately without further preparation into a sterile Falcon tube, representing the state after procurement. A second sample cube was prepared by removing the corticalis according to standard operating procedures at the DIZG before being transferred into a sterile Falcon tube. These two sample cubes were refrozen at < − 18 °C. The third sample cube was sent to the tissue bank of the Charité Berlin, where chloroform treatment for defatting was performed. This sample cube was then returned to the DIZG and stored at < − 18 °C as well.

Lastly, all sample cubes were sent to the contracted and accredited laboratory for bioburden examination. During all transports, the sample cubes were stored on dry ice.

### Bioburden examination

Bioburden was respectively determined after procurement, mechanical preparation, and chloroform (CHCI3) treatment for defatting at the tissue bank of the Charité, Berlin.

The determination of the bioburden included both the total number of bacteria (TAMC, Total Aerobic Microbial Count) and the number of yeasts and moulds (TYMC). The methodological specifications for the investigation were consistently transferred to the TAMC anaerobic parameters and the quantification of *Enterococcus*. Bioburden determination was performed in accordance with the European Pharmacopoeia, Chapter 2.6.12 Microbiological testing of non-sterile products: Counting the number of microorganisms capable of reproduction (European Pharmacopoeia 2022).

#### Equipment, culture media, incubation temperature and time

See Tables 1 and 2.

**Table 1:** Overview of the culture media

Culture media	Manufacturer	Article no	Application
Sojapepton-Caseinpepton-Agar (CASO-Agar, TSA, EP harm.)	sifin diagnostics gmbH (Berlin, DE)	TN1031	TAMC-aerob/anaerob
Sabouraud-Dextrose-Agar (SABO-Agar, SDA, EP harm.)	sifin diagnostics gmbH (Berlin, DE)	TN1264	TYMC
Galle-Aesculin-Azid-Agar (BAA-Agar)	Oxoid Deutschland GmbH (Wesel, DE)	P05062A	*Enterococci*

**Table 2:** Materials used to set anaerobic incubation conditions

Material	Manufacturer	Article no
Anaerobic jar	Merck KGaA (Darmstadt, DE)	116387
Anaerogen	Thermo Fisher Scientific Inc. (Waltham, Massachusetts, USA)	AN0025A
Anaero-Teststrips	Merck KGaA (Darmstadt, DE)	1.15112
NaCl-Pepton Puffer	sifin diagnostics gmbH (Berlin, DE)	P05062A

#### Preparation of sample solution from the iliac crest tissue samples

The preparation of sample suspensions was carried out with a sodium chloride-peptone buffer solution (NaCl-PP) with a pH value of 7. As an additive 0.1% Tween 80 (E8) was used, a surface-active substance that facilitates suspension. 10 g of the partially processed tissue sample were suspended homogeneously in 100 ml of NaCl-PP + E8 by shaking manually, so that a sample solution with a ratio of 1:10 is created.

##### Sample preparation

See Table 3.

**Table 3:** Overview preparation of the plate cultures

	Sample volume	Method	Culture medium	Incubation time
TAMC-aerob	2 × 1 ml	Plate pour method	CASO-Agar	30–35 °C for 3–5 days
TAMC-anaerob	2 × 1 ml	Plate pour method	CASO-Agar	30–35 °C for 3–5 days under anaerobic conditions
TYMC	2 × 1 ml	Plate pour method	SABO-Agar	20–25 °C for 5–7 days
*Enterococci*	4 × 500 µl or: Further decadal solutions in NaCl-PP + E8	NaCl-PP + E8	NaCl-PP + E8	NaCl-PP + E8

#### Count of CFU/g

To determine the CFU/g for the TAMC aerobic and anaerobic, all colonies on the CASO agar were counted. Analogously, all colonies on the SABO agar were counted for the TYMC. To determine the number of *Enterococci*, all gray-black colonies with a dark halo on BAA agar were counted.

The lower limit of detection was 10 colony forming units per gram tissue (CFU/g) for all assays. For statistical analysis samples without growth (< 10 CFU/g) were transferred as 0 CFU/g into the data set.

The acceptance criterion for this study was defined as 9 × 10^4^ CFU/g tissue, as this is below the validated capacity of the peracetic sterilization process at the DIZG (M. Löwel, personal communication, 01.06.2024).

## Results

**Fig. 1:**
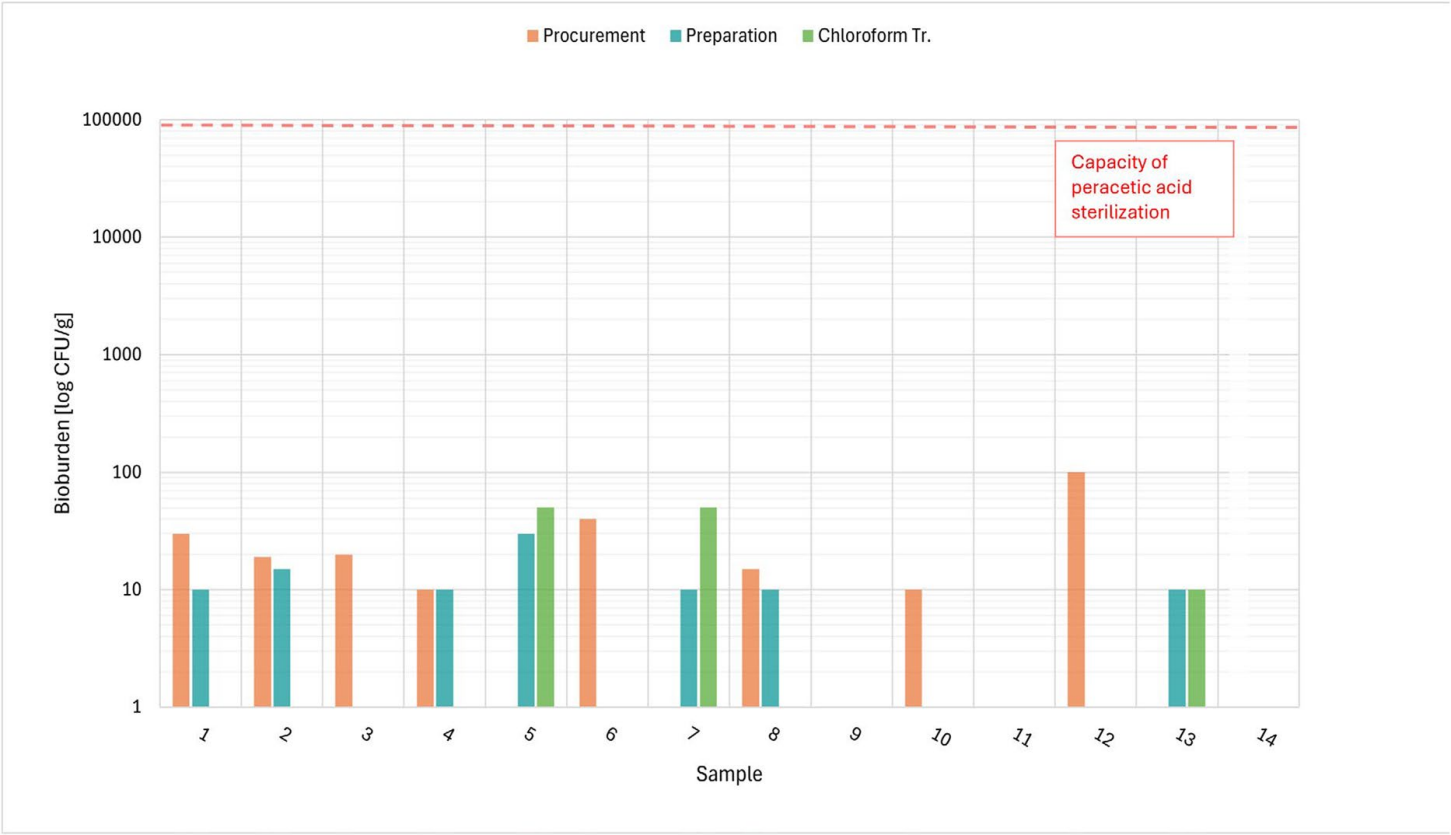
TAMC aerobe: CFU/g in 14 cases after 48 h and respectively after procurement, preparation and chloroform treatment

**Fig. 2:**
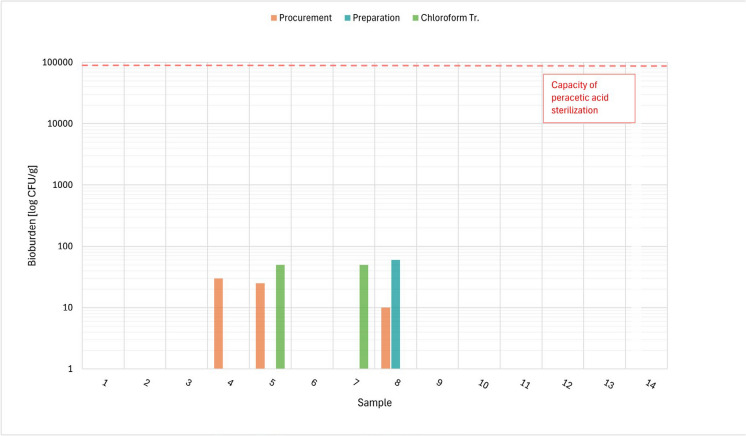
TAMC anaerobe: CFU/g in 14 cases after 48 h and respectively after procurement, preparation and chloroform treatment

**Fig. 3:**
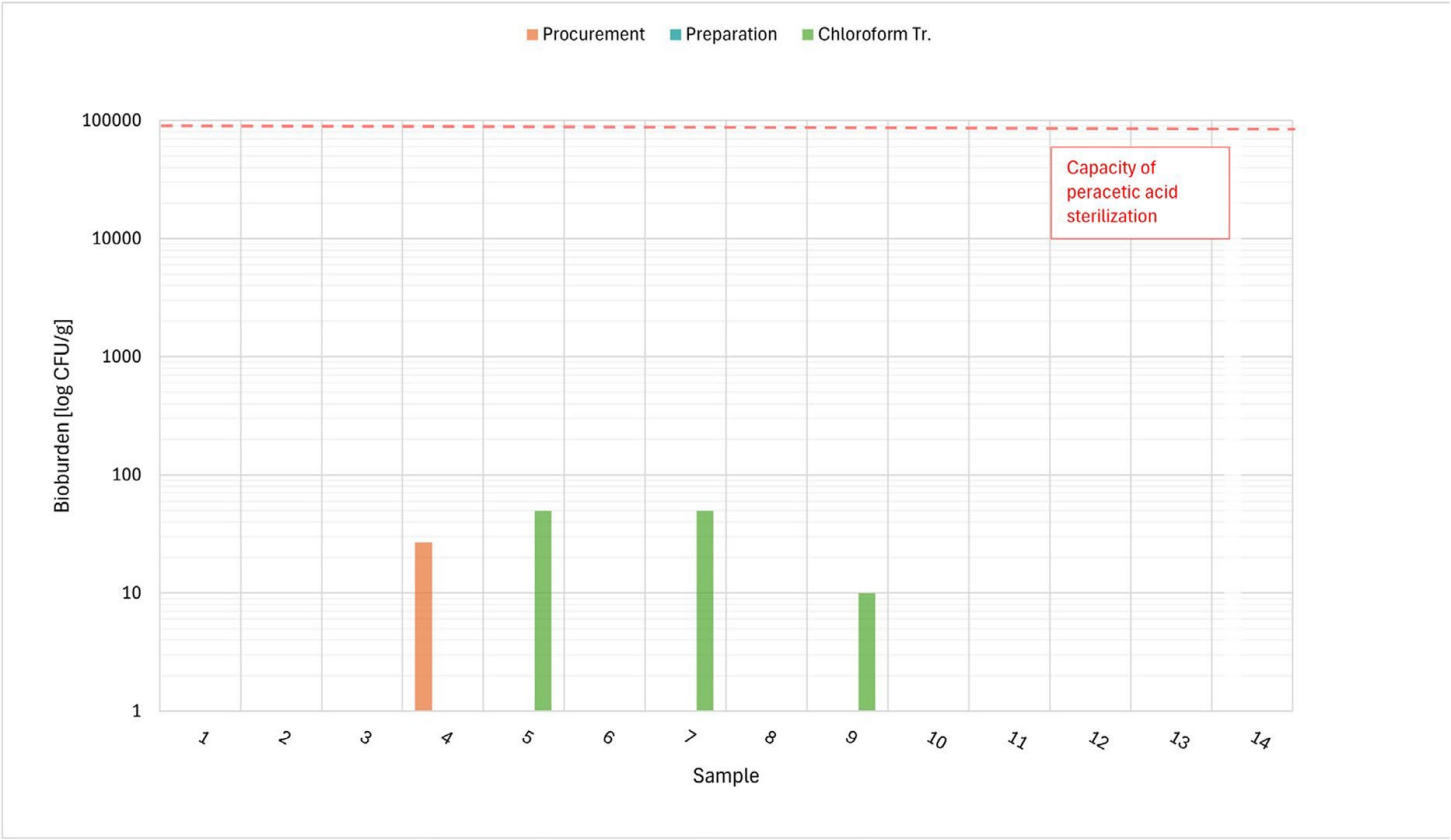
TYMC: CFU/g in 14 cases after 48 h and respectively after procurement, preparation and chloroform treatment

**Fig. 4:**
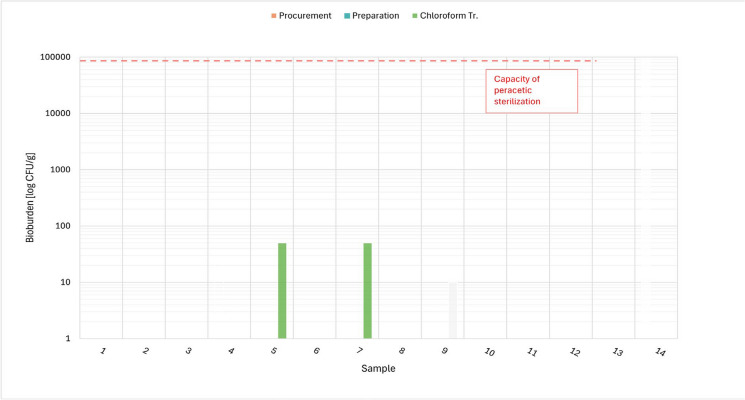
*Enterococcus*: CFU/g in 14 cases after 48 h and respectively after procurement, preparation and chloroform treatment

The results of the bioburden examination concerning the total aerobic and anaerobic microbial count, total yeast/moulds count and *Enterococcus* are presented in Tables 5, 6, 7. Figures 1, 2, 3 and 4 show the total values of the bioburden for group 1 (procurement within 48–54 h). We found across all examinations 29/168 (17.3%) positive samples in group 1 and 10/84 (11,9%) positive samples in group 2 (procurement within 72–78 h). The control group with 4 samples had no positive findings. However, all positive bioburden findings were below the capacity of the peracetic sterilization process.

In Table 4 the age distribution of all 3 groups and the postmortem intervals until procurement are presented with mean values, standard deviation (SD) and range.

**Table 4:** Donor age distribution and postmortem interval until procurement

		Group 1 (48 h) n = 14	Group 2 (72 h) n = 7	Group 3 Control (36 h) n = 4
Age (years)	Mean (± SD)	67,5 (15,9)	66,0 (19,4)	78,0 (3,7)
	Range	40–86	22–80	73–82
Procurement (hours)	Mean (± SD)	49,9 (2,2)	73,6 (1,8)	24,5 (4,0)
	Range	46–53,5	72–76	18–29

### Total aerobic microbial count

Table 5 shows the mean number and standard variation as well as the range and number of cases without contamination after procurement, preparation, and chloroform treatment for the total aerobic microbial count of all three groups. Figure 1 shows the results of CFU/g tissue per individual case in group 1 (48 h).

**Table 5:** Total aerobic microbial count in CFU/g ± SD of iliac crest bone graft tissue sample obtained after different postmortem time intervals during procurement, preparation and defatting steps

Step	TAMC aerobic	Group 1 (48 h) n = 14	Group 2 (72 h) n = 7	Group 3 Control (36 h) n = 4
Procurement	Mean (± SD)	17.4 (26.0)	31.4 (61.0)	0.0 (0)
	Range	0–100	0–180	0.0
	Cases with CFU 0 (%)	6/14 (42.9%)	3/7 (42.9%)	4/4 (100%)
Preparation	Mean (± SD)	6.8 (8.4)	45.0 (85.7)	0.0 (0.0)
	Range	0–30	0–250	0
	Cases with CFU 0 (%)	7/14 (50%)	4/7 (57.1%)	4/4 (100%)
Chloroform treatment	Mean (± SD)	7.9 (17.4)	1.4 (3.5)	0.0 (0.0)
	Range	0–50	0–10	0
	Cases with CFU 0 (%)	11/14 (78.6%)	6/7 (85.7%)	4/4 (100%)

In group 1, the highest contamination was detected after procurement with 100 CFU/g, after preparation with 30 CFU/g and after chloroform treatment with 50 CFU/g. Six cases in this group did not show any growth after procurement, 7 after preparation and 11 after chloroform treatment.

In group 2, the highest contamination was detected after procurement with 180 CFU/g, after preparation with 250 CFU/g and after chloroform treatment with 10 CFU/g. Three cases in this group did not show any growth after procurement, 4 after preparation and 6 after chloroform treatment.

### Total anaerobic microbial count

Table 6 shows the results for the total anaerobic microbial count, Fig. 2 the results of CFU/g tissue per individual case for group 1 (48 h).

**Table 6:** Total anaerobic microbial count in CFU/g ± SD of iliac crest bone graft tissue sample obtained after different postmortem time intervals during procurement, preparation and defatting steps

Step	TAMC anaerobic	Group 1 (48 h) n = 14	Group 2 (72 h) n = 7	Group 3 Control (36 h) n = 4
Procurement	M (± SD)	4.6 (9.7)	30.0 (73.5)	0 (0.0)
	Range	0–30	0-210	0
	Cases with CFU 0 (%)	11/14 (78.6%)	6/7 (85.7%)	4/4 (100%)
Preparation	M (± SD)	4.3 (15.5)	0 (0.0)	0 (0.0)
	Range	0–60	0	0
	Cases with CFU 0 (%)	13/14 (92.9%)	7/7 (100%)	4/4 (100%)
Chloroform treatment	M (± SD)	7.1 (17.5)	0 (0.0)	0 (0.0)
	Range	0–50	0	0
	Cases with CFU 0 (%)	12/14 (85.7%)	7/7 (100%)	4/4 (100%)

In group 1, the highest contamination was detected after procurement with 30, after preparation with 60 and after chloroform treatment with 50 CFU/g. 11 cases in this group did not show any growth after procurement, neither did 13 after preparation and 12 after chloroform treatment.

Cases 5 and 7 in group 1 did show 50 CFU/g after chloroform treatment in group 1, however we did not see any positive findings during the preceding steps in one of those cases and only 25 CFU/g after procurement and no findings after preparation in the other one. In both these cases we also found 50 CFU/g tissue for TAMC, TYMC and *Enterococcus* after this step (see Figs. 1, 3, 4 and below).

In group 2, the highest contamination was detected after procurement with 210 CFU/g, the 6 other cases did not show any growth. After preparation and chloroform treatment no growth was detectable at all.

### Total combined yeasts/moulds count

Table 7 shows the results for the total combined yeasts/moulds count, Fig. 3 the results of CFU/g tissue per individual case for group 1 (48 h).

**Table 7:** Total yeast/mould count in CFU/g ± SD of iliac crest bone sample obtained after different postmortem intervals during procurement, preparation and defatting steps

Step	TYMC	Group 1 (48 h) n = 14	Group 2 (72 h) n = 7	Group 3 Control (36 h) n = 4
Procurement	M (± SD)	1.9 (7.0)	24.3 (59.5)	0 (0.0)
	Range	0–27	0–170	0
	Cases with CFU 0 (%)	13/14 (92.9%)	6/7 (85.7%)	4/4 (100%)
Preparation	M (± SD)	0 (0.0)	0 (0.0)	0 (0.0)
	Range	0	0	0
	Cases with CFU 0 (%)	14/14 (100%)	7/7 (100%)	4/4 (100%)
Chloroform treatment	M (± SD)	7.9 (17.4)	0 (0.0)	0 (0.0)
	Range	0–50	0	0
	Cases with CFU 0 (%)	12/14 (85.7%)	7/7 (100%)	4/4 (100%)

In group 1, the highest contamination was detected after procurement with 27 and after chloroform treatment with 50 CFU/g (see below). No growth was detected after preparation. After procurement the other 13 cases did not show growth, as did 12 cases after chloroform treatment.

In group 2, the highest contamination was detected after procurement with 170 CFU/g. The remaining 6 cases did not show any growth. The same is true for all samples after preparation and chloroform treatment.

#### Enterococcus

In neither of the groups *Entercoccus* was detected after procurement and preparation. Again, cases 5 and 7 in group 1 detected *Enterococcus* with 50 CFU/g after chloroform treatment (Fig. 4) with all other results negative. However, in the preceding steps no *Enterococcus* could be detected. As presented above, these two cases did show 50 CFU/g across all the examinations after chloroform treatment.

In our opinion, this strongly suggests a probable handling mistake for cases 5 and 7 of group 1 in the microbiological lab. This is compounded by the fact that both samples were incubated during the same point of time.

## Discussion

The aim of tissue donation is to provide high-quality transplants that are safe for the recipient. There are corresponding requirements concerning the safety and quality of tissues transplants in national legislation and in the European Union (Doerr 2022; EU-Directive 2004/23/EC).

The EDQM Guide defines that procurement can be carried out aseptically or under 'clean' conditions if a validated sterilization procedure ensures safety. Under these circumstances, a collection time of up to 48 h postmortem is considered possible (Doerr 2022).

Several studies have been carried regarding the microbial contamination and factors that increase the risk of such an occurrence. In addition to a longer PMI until procurement, factors found to contribute to a contamination of explanted tissues are a lengthy procurement procedure (Brubaker et al. 2016; Bohatyrewicz et al. 2006; Viñuela-Prieto et al. 2019) and the number of staff performing the procurement (Brubaker et al. 2016; Paolin et al. 2017a; Bohatyrewicz et al. 2006; Vehmeyer et al. 2002; Terzaghi et al. 2015; Deijkers et al. 1997). Also, bone allografts seem to show higher contamination rates than tendon grafts (Terzaghi et al. 2015).

The risk also seems to be greater for NHBD in comparison to multiorgan donors (MOD) (Bohatyrewicz et al. 2006; Paolin et al. 2017a, b), as did procurement in a morgue (Bohatyrewicz et al. 2006). However, other studies found that procurement in a morgue may also reduce the risk of contamination, probably due to lower temperatures when compared to a standard operating theatre (Bettin et al. 1998; Louart et al. 2019). Journeaux et al. (1999) reported that NHBD hemipelvis procured in a morgue has a higher risk of contamination, but not femur and tibia when compared to MOD. Paolin et al. (2017b) found that MST are predominantly contaminated by one single bacterial strain, multiple strains are detected in only about 10% of the tissues. In their study, these strains are mainly attributed to skin commensals, which is supported by Deijkers et al. (1997), who also found these strains to be of low pathogenicity. Lastly, Kumta et al. (1997) found that cadaveric bone allograft in a rat model could be explanted under sterile conditions up to 96 h after death.

Concerning contamination in postmortem blood samples, Ibrahim et al. (2004) did not find any significant differences in samples taken under and over 24 h, while Dolan et al. (1971) reported that the incidence of positive cultures with cadaveric tissue does not increase with postmortem time up to 48 h after death.

Overall, exogeneous causes of contamination (e.g. handling of the donor body, techniques used during procurement etc.) can be reduced by the procurement team (Brubaker et al. 2016; Paolin et al. 2017a; Terzaghi et al. 2015; Deijkers et al. 1997). Endogenous causes of contamination can be controlled by donor selection and a short postmortem interval (Brubaker et al. 2016; Paolin et al. 2017a; Vehmeyer et al. 2002; Terzaghi et al. 2015).

For our study we used standard procurement techniques to reduce the risk of contamination during procurement. We chose the iliac crest as target tissue, with procurement after exposing the ilium according to standard hemipelvis explantation. We postulate that this tissue has a higher risk of contamination due to its proximity to the intestine, as is supported by the findings of Journeaux et al. (1999) and as such serving as a ‘worst-case scenario’ for MST with a postmortem procurement time of 48 and 72 h respectively.

The main limitation of our study is the sample size, which can be explained by the difficulties in recruiting donors: For the control group, only cases with contraindications to donation (like age, carcinoma or dementia) may be used for the study. For group 1 and especially 2, donors must arrive at our institute within 6 h and stay there without an autopsy up to 48/72 h for a procurement procedure during working hours.

Notwithstanding, several conclusions can be drawn from our results:

Comparing the control group (without any positive findings) with either group 1 or 2, the data support the fact that contamination rates increase with time. This is especially obvious for the total aerobic microbial count. Here we saw in group 1 a contamination in at least one of the examination steps of 8/14 cases after procurement, 7/14 cases after preparation and in 3/14 cases after chloroform treatment. In group 2 contamination was seen in 4/7 cases after procurement, 3/7 cases after preparation and in 1/7 cases after chloroform treatment.

However, daily experience in the tissue bank of the DIZG shows that contamination rates comparable to groups 1 and 2 may be found in bone tissues procured under non-strict-aseptic procurement conditions within 24 and 36 h (Pruss et al. 2001).

Our results show clearly that none of the positive samples exceeded a maximal bacterial or fungal bioburden of 250 CFU/g tissue (group 2, total aerobic microbial count). All samples were far below the acceptance criterion of 9 × 10^4^ CFU/g tissue.

In conclusion we show that the bioburden of our samples with longer procurement times are well within the limit of the capacity of the sterilization process at the DIZG.

On basis of our data, we suggest that an extension of the procurement period up to 48 h seems feasible, if the bioburden is determined on a regular basis and the tissues are sterilized by a validated process, as suggested by Eastlund (Eastlund 2006). Indeed, after presentation of all data to the Paul-Ehrlich-Institute, the responsible competent authority in Germany concerning tissue transplants, a collection time of up to 48 h was granted. Further studies are necessary to investigate a possible procurement time up to 72 h.

## Data Availability

No datasets were generated or analysed during the current study.
